# Litter input decreased the response of soil organic matter decomposition to warming in two subtropical forest soils

**DOI:** 10.1038/srep33814

**Published:** 2016-09-20

**Authors:** Qingkui Wang, Tongxin He, Jing Liu

**Affiliations:** 1 Key Laboratory of Forest Ecology and Management, Institute of Applied Ecology, Chinese Academy of Sciences, Shenyang 110164, P. R. China; 2 Huitong Experimental Station of Forest Ecology, Chinese Academy of Sciences, Huitong 418307, P.R. China; 3 University of Chinese Academy of Sciences, Beijing 100049, P.R. China

## Abstract

Interaction effect of temperature and litter input on SOM decomposition is poor understood, restricting accurate prediction of the dynamics and stocks of soil organic carbon under global warming. To address this knowledge gap, we conducted an incubation experiment by adding ^13^C labeled leaf-litter into a coniferous forest (CF) soil and a broadleaved forest (BF) soil. In this experiment, response of the temperature sensitivity (*Q*_10_) of SOM decomposition to the increase in litter input was investigated. The temperature dependences of priming effect (PE) and soil microbial community were analyzed. The *Q*_10_ for CF soil significantly decreased from 2.41 in no-litter treatment to 2.05 in litter-added treatment and for BF soil from 2.14 to 1.82, suggesting that litter addition decreases the *Q*_10_. PE in the CF soil was 24.9% at 20 °C and 6.2% at 30 °C, and in the BF soil the PE was 8.8% at 20 °C and −7.0% at 30 °C, suggesting that PE decreases with increasing temperature. Relative PE was positively related to the concentrations of Gram-negative bacterial and fungal PLFAs. This study moves a step forward in understanding warming effect on forest carbon cycling by highlighting interaction effect of litter input and warming on soil carbon cycling.

Soil in terrestrial ecosystems contains 1550 Pg organic C^1^, which is a result of the balance between input from leaves and output from decomposition of soil organic matter (SOM)^2^. SOM decomposition is not only controlled by temperature and moisture^2^ but also affected by litter input^3^. The global increase of 1.1–6.4 °C in temperature by the end of the 21st century is likely to alter the input of litter to soil systems^4^,^5^. This alteration of exogenous substrate input will change soil organic C (SOC) stocks through priming effect (PE)^6^. In addition, changes in the temperature sensitivity (*Q*_10_) of SOM decomposition induced by increasing substrate input also determine the response of terrestrial C balance to global warming^7^. Therefore, improving prediction of SOC dynamics under global warming is important to understand the combined effect of temperature and litter input on SOM decomposition.

Unfortunately, a consensus has not yet been reached on the magnitude of *Q*_10_ of SOM decomposition because of a large variation on *Q*_10_, although considerable efforts have been exerted to study *Q*_10_ values^2^,^8^,^9^. This inconsistency may stem from confounding factors such as substrate availability and microbial community structure^10^,^11^. Increased litter input to soil systems is likely to alter substrate availability by releasing labile C during litter decomposition. Recently, a number of empirical studies have identified the control of labile substrate availability to *Q*_10_ of microbial respiration^11^,^12^,^13^; these studies demonstrated that the addition of readily available C substrate significantly increased *Q*_10_ values. Despite its importance, substrate availability is probably the least studied factor that affects the *Q*_10_ of SOM decomposition^2^. The abovementioned studies generally used glucose as sources of labile substrate. However, as an important source of labile substrate in actual forest ecosystems, how litter addition affects *Q*_10_ still remains poor understood.

Another important process that affects SOC cycling is PE, being defined as the changes in the decomposition rate of SOM after input of exogenous substrates^14^. Several studies on PE have been conducted, but the direction and intensity of PE in various experiments are large uncertain, which range from −95.1% to 1207%^15^,^16^. This uncertainty is attributed to the fact that PE is dependent on many factors, such as incubation temperature^17^,^18^, soil nutrient availability^19^,^20^,^21^, and characteristics of soil microbial community^9^. For example, Kuzyakov predicted that incubation temperature may mediate PE because increasing temperature can accelerate most enzyme activities^17^; however, direct evidence is lacking on the influence of temperature on the PE in forest ecosystems, although some studies reported the impacts of temperature on rhizosphere PE in arable soils^22^,^23^. Therefore, it remains unclear whether SOC losses through PE would change after temperature increase.

The important role of biotic factors (e.g., microbial community structure) in SOM decomposition is now being recognized^17^,^20^,^24^. The alteration of soil microbial community structure and activity by exogenous substrate addition has been demonstrated^20^,^25^, which may affect *Q*_10_^13^ and PE^26^,^27^. Some studies found that the level of microbial community response affected *Q*_10_^9^. In addition, a shift from C limitation for soil microorganisms to nutrient limitation induced by exogenous C addition may result in microbial mining additional SOM to acquire nutrients, and therefore positive PE^20^,^28^. However, how the response of soil microbial community structure to temperature affects *Q*_10_ and the PE of SOM decomposition remains unclear.

In the present study, we presented the result of an incubation experiment that combined short-term manipulations of temperature and substrate (^13^C-labeled leaf litter) addition in two different soils from a coniferous forest (CF) and an evergreen broadleaved forest (BF). The ^13^C isotopic differences between litter and native SOC allowed the quantification of CO_2_-C sources from decomposition of litter and SOM. These data were used to determine *Q*_10_ and PE in different soils. The present study aimed to investigate the interactive effect of exogenous substrate input and warming on SOM decomposition in subtropical forests. Based on our knowledge, we hypothesized that litter input would decrease *Q*_10_, and warming would decrease PE intensity.

## Results

### Soil microbial community composition

Litter addition increased the common bacterial concentration by 24.4% and 26.8% in CF soils and 11.0% and 15.4% in BF soils at 20 and 30 °C, respectively (Table 1). Fungal concentration was increased by 54.6% and 37.5% in CF soils and 26.6% and 17.9% in BF soils at 20 and 30 °C, respectively. However, litter addition increased Gram-positive bacterial concentrations averagely by 14.2% in two soils only at 30 °C and increased Gram-negative bacterial concentrations only in the CF soils by 22.9% and 17.6% at 20 and 30 °C, respectively. Litter addition decreased the ratio of bacteria to fungi with an average of 18.4%, with the exception of BF soil at 30 °C. Increasing temperature increased bacterial concentrations averagely by 15.8% and the ratio of bacteria to fungi averagely by 22.4% in BF soils. Compared with CF soils, BF soils had higher microbial concentrations measured by phospholipid fatty acids (PLFAs) but lower than the ratios of Gram-positive to Gram-negative bacteria and bacteria to fungi at the same temperature.

### Litter-C fate

**Figure 1:**
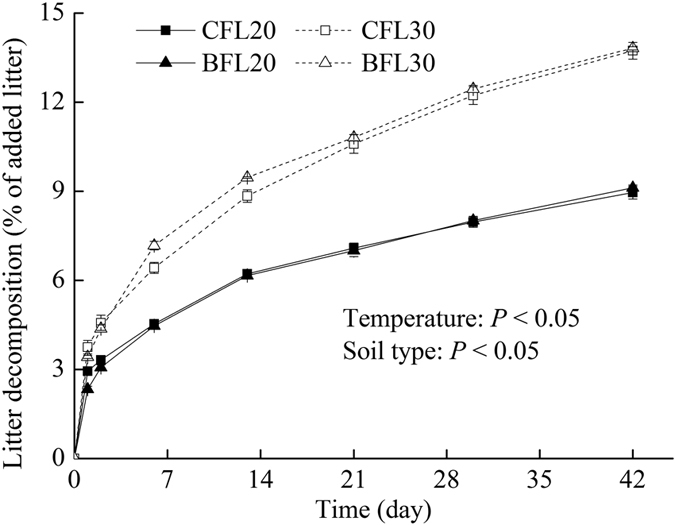
The cumulative fraction of added litter C released as CO_2_ during the 42-day incubation period.

Expressed as percentage of the amount of added litter, CO_2_ released rate from litter differed among treatments (Fig. 1). However, the pattern of litter decomposition was similar among all treatments. Increasing temperature promoted litter decomposition averagely by 52.4%. However, litter decomposition between in CF and BF soils did not differ. At the end of incubation, approximately 10% of the added litter-C was respired as CO_2_ at 20 °C and 12.7% at 30 °C for both soils.

**Figure 2:**
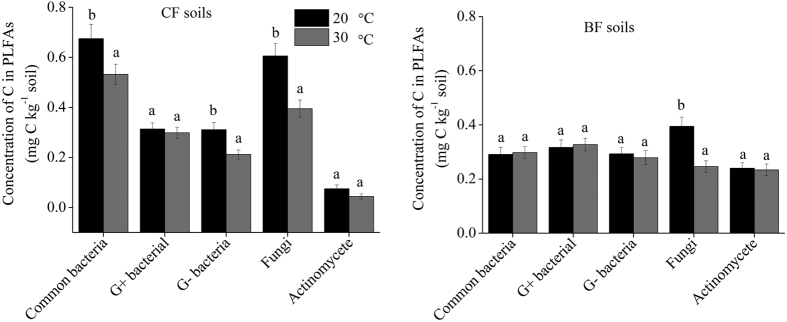
Concentration of litter-derived C within different PLFAs 42 days after treatments. Significant differences (*P* < 0.05) are labeled by asterisks between treatment pairs (t-test). Data are means with SD (*n* = 3).

The amount of litter-derived C incorporated into PLFAs was affected by temperature and soil type (Fig. 2). Over the 42-day incubation, the litter-C incorporated into total PLFAs was 1.98 mg C kg^−1^ and 1.48 mg C kg^−1^ soil for CF soil at 20 °C and 30 °C, respectively, and 1.53 mg C kg^−1^ and 1.24 mg C kg^−1^ for BF soil. Elevating temperature decreased the amount of litter-C incorporated into fungal PLFAs in the both soils, as well as the common and Gram-negative bacterial PLFAs in the CF soil.

### Temperature sensitivity of SOM decomposition

**Figure 3:**
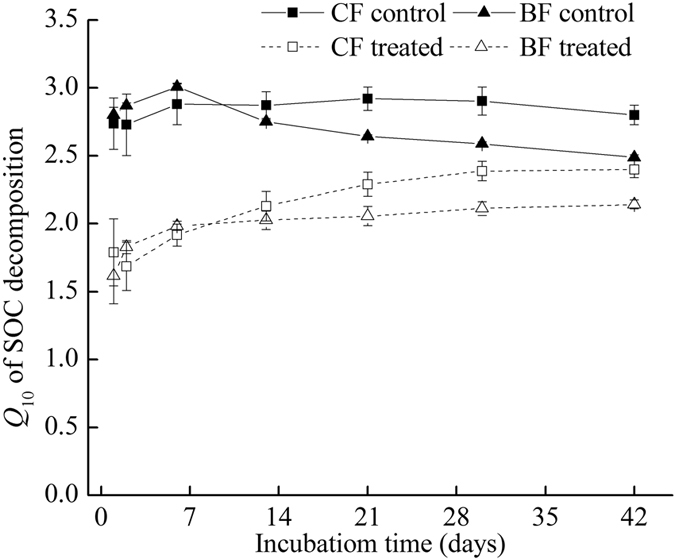
Temperature sensitivity (*Q*_10_) of SOM and litter decomposition in the control (no litter) and treated (added litter) soils from a coniferous forest (CF) and a broadleaved forest (BF) during the 42-day incubation period.

The temperature sensitivity of SOM decomposition in the treatments without litter addition was significantly different in the CF and BF soils (Fig. 3). CF soils (2.41 ± 0.10) had slightly higher *Q*_10_ value than BF soils (2.14 ± 0.13) at the end of the 42-day incubation. A significant decline in *Q*_10_ was detected after litter addition, and *Q*_10_ decreased to 2.05 in the CF soils and 1.82 in the BF soils, indicating that litter addition decreased the response of SOM decomposition to warming. The *Q*_10_ dynamics in the CF soils without litter remained constant, but *Q*_10_ in the BF soils without litter initially increased slightly and then decreased significantly. By contrast, litter addition led to a significant increase in the *Q*_10_ with time, indicating that litter addition modifies the dynamics of the temperature sensitivity of SOM decomposition.

### Priming effect

**Figure 4:**
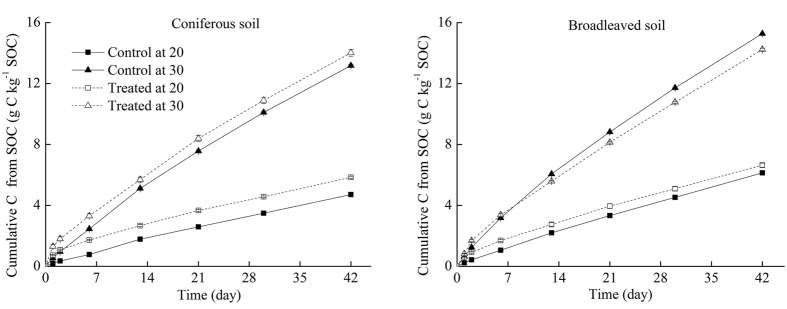
Cumulative C respired as CO_2_ from SOM in the control soils and litter-amended soils at 20 and 30 °C from a CF and a BF during the 42-day incubation period.

The cumulative CO_2_–C released from SOM in the control at 30 °C was 140% higher for CF soils and 113% higher for BF soils than their counterparts at 20 °C (Fig. 4). After adding litters, the CO_2_–C that respired from SOM at 30 °C was 104% and 82% higher for CF and BF soils than that at the 20 °C. These results indicate that litter addition decreases the response of SOM decomposition to temperature elevation. In the control, the SOM decomposition of BF soils was 30% greater than that of CF soils at 20 °C, and 16% higher at 30 °C, whereas in the litter-added treatments, the SOM decomposition of BF soils was 14% greater than that of CF soils at 20 °C, and 2% higher at 30 °C.

**Figure 5:**
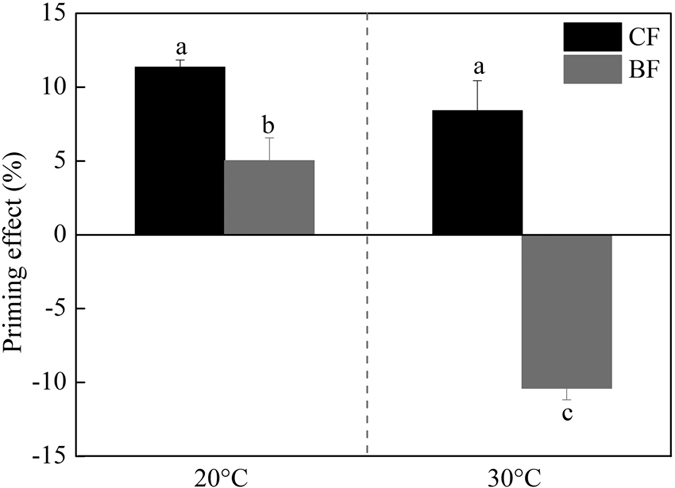
The relative priming effect of litter addition on SOM decomposition in soils from a CF and a BF at 20 and 30 °C.

Litter addition primed SOM decomposition, but relative PE differed in CF and BF soils (Fig. 5). PE was higher in the CF soils (24.9%) than in the BF soils (8.8%) at 20 °C. Stimulation effect (positive PE = 6.2%) was observed in the CF soils at 30 °C, but inhibition effect (negative PE = –7.0%) was found in the BF soils. This result indicates that elevating temperature decreases PE in both soils at the same degree. A significantly positive correlation was observed between relative PE and the concentrations of fungi and Gram-negative bacteria PLFAs, and a negative correlation was found between relative PE and the ratio of bacteria to fungi in both forest soils (Table 2).

## Discussion

To the best of our knowledge, this study is the first to investigate the effect of litter input on the temperature sensitivity of SOM decomposition in forest ecosystems. The finding that litter addition decreased the temperature sensitivity of SOM decomposition supported our first hypothesis; however, this finding contradicts the observations of some studies that suggested that the addition of readily available carbon substrate increased *Q*_10_^10^,^12^,^13^. This different response may be attributed to the difference in the quality of substrates added to the soils. The litter used in our experiment was recalcitrant to soil microorganisms, whereas in other studies sucrose or glucose was used. Another potential explanation is the fact that the water-soluble N in the litter entered the soils and thereby increased soil N availability, resulting in a low response of SOM decomposition to elevated temperature. Decline in the *Q*_10_ of SOM decomposition after litter addition suggests that the stimulation effect of warming on SOM decomposition will be decreased by the increase in litter input under atmospheric CO_2_ enrichment and can partly abate C losses in this subtropical region. Therefore, this will result in a less positive feedback to climate change than previously expected for subtropical forest ecosystems.

Soil microbial community is generally limited by C^29^, and some studies documented that an increase in substrate availability positively affects temperature sensitivity^10^,^13^. In the present study, the significant increase in the *Q*_10_ of soils with litters during incubation was attributed to the gradual release of dissolved organic substrate in litters to the soil, which provided energy for soil microorganisms. In the BF soils without litter, a decrease in *Q*_10_ with incubation time was also attributed to the decrease of substrate availability because of the depletion of liable substrate in soils.

Regardless of the litter addition, higher *Q*_10_ was observed in the CF soils than in the BF soils. This result may be explained by the higher C:N ratio of the CF soil than that of the BF soil (Table 3) because low-quality SOM with high C:N ratio has higher temperature sensitivity than high-quality SOM with low C:N ratio^30^. Some studies also found that SOM quality influences the temperature sensitivity of SOM decomposition^31^,^32^. CF soils also had a lower N content than BF soils (Table 3), which could result in significant N depletion with increasing temperature and potentially high SOM decomposition. In the present study, CF soils had higher ratios of bacteria:fungi and Gram-positive:Gram-negative bacteria than BF soils (Table 1). The pine litter used in the present study was more compositionally similar to the litter from CF than BF. Thus, in comparison with BF soil, the microbes that decompose pine litter would be more likely to use CF soil as C source under elevated temperature in this forest ecosystem, which increased SOM mined by microbes to gain additional N in CF soil.

An important finding in our study is the strong decrease in PE with increasing soil temperature in the subtropical forest ecosystem. This finding indicates that the magnitude of PE depends on environmental temperature. Our findings confirmed the prediction of Kuzyakov using experimental data^17^. In a meta-analysis literature, Zhang *et al.* also found that PE intensity below 20 °C (38.4%) was significantly higher than that above 20 °C (20.2%)^16^. One potential mechanism for this phenomenon is the fact that fast growth of microbes at high temperature could lead to more N immobilization^33^. Another potential explanation is the high SOM decomposition level in soils without litter at high temperature (Fig. 4). A similar observation was also reported by Thiessen *et al.*^9^.Our findings indicate that global warming will likely decrease the effect of PE induced by litter input on SOC storage in the subtropical forests. The response of PE to temperature change is crucial in predicting the response of SOM decomposition in future climate change. Therefore, additional experiments on this topic should be widely conducted in a range of forest ecosystems in future research.

A higher PE was observed in CF soils than in BF soils (Fig. 5), indicating that external C input will result in higher native SOC loss in CF soils than in BF soils. According to discussions of Zhang *et al.*^16^, differences in soil properties (e.g., NH_4_^+^N content, C:N ratio) can partially explain PE variations. Two mechanisms may explain these results. The first is that low N availability stimulates soil microbes to mine SOM and search for nutrients to sustain their growth^14^,^34^. In the present study, BF soils had richer amounts of nutrients (e.g., N, P) and lower C:N ratio than CF soils (Table 3), which result in lower PE in BF soils than that in CF soils. In a temperate forest, Wang *et al.* demonstrated that soils with high C:N ratio had high PE after soluble C addition^35^. The second mechanism is the concept of the “nutrient bank” mechanism proposed by Fontaine and Barot^36^, wherein microbial N mining regulates SOM decomposition and nutrient release. This mechanism is supported by the negative relationship between PE and nutrient availability^37^,^38^.

PE also depends on the characteristics of soil microbial community^9^,^17^,^28^. In the present study, soil microbial growth and changes in community structure occurred after litter input (Table 1), which were partly responsible for PEs. Recently, some studies also suggested that PE may be related to soil microbial community because PEs are accompanied by changes in the composition of soil microbial community^20^,^39^,^40^; and Bell *et al.* showed that PE was related to the size of biomass and its composition, particularly bacteria:fungi ratio^26^. In the present study, significantly positive relationships were observed between fungi, Gram-negative bacteria and PE (Table 1), suggesting that fungi and Gram-negative bacteria play an important role in PE. This result also confirms previous observations that Gram-negative bacteria or fungi may be particularly important in metabolizing soil SOM^37^,^39^,^41^.

In conclusion, litter input decreases the temperature sensitivity of SOM decomposition. This finding suggests that, in the context of global warming, stimulation of increasing temperature on SOM decomposition is likely to be decreased by increased litter input under elevated atmospheric CO_2_ in the subtropical China. Litter addition induces PE, but PE magnitude decreases with elevated temperature. Thus, acceleration of global warming on SOM decomposition may be partly compensated by the decrease in PE with increasing temperature in subtropical forest ecosystems. Significant correlations between PE and the concentrations of Gram-negative bacterial and fungal PLFAs indicate that Gram-negative bacteria and fungi may have important functions in metabolizing SOM and PE. To better understand and model influence of global change on SOC cycle, an accurate evaluation of the sources of primed C from recent and old SOM pools is required in future.

## Material and Methods

### Study sites and soil sample preparation

The soil samples were collected from a CF and an evergreen BF at Huitong National Research Station of Forest Ecosystem (26°40′N, 109°26′E) in Huitong County, Hunan Province, China. The mean annual temperature (1998 to 2014) was 16.5 °C and the mean annual rainfall was 1200 mm to 1400 mm. The soil derived from shale is classified as Ultisols according to the second edition of U.S. Soil Taxonomy. The CF is a monoculture plantation of *Cunninghamia lanceolata* (about 26 years old), and the BF is dominated by *Cyclobalanopsis glauca*, *Machilus pauhoi*, and *Liquidam bar formosana*. Mineral soil was randomly sampled from 0 cm to 10 cm depth after removing organic matter layer. Fresh soil was immediately transported to the laboratory and sieved through a 2-mm mesh sieve. The remaining organic residues and stones were carefully removed by hand from the soils. The physicochemical properties of the two soils are shown in Table 3.

### Experiment design and CO_2_ measurement

All soil samples were pre-incubated for one week before incubation, and then divided into two temperature groups (20 and 30 °C). A trace technique was used to label tree seedling in a growth chamber^42^,^43^. The seedlings were continuously labeled for three months with ^13^CO_2_ gas with an abundance of 99.9 atom%. In this incubation experiment, we used the needles that newly emerged during labeling to insure uniform ^13^C distribution among the structural and metabolic litter components. In order to test whether the needles were uniformly labeled, ground needles were extracted with hot-water (80 °C) for 16 h with three replicates, and then the δ^13^C of extract and residues were measured. The δ^13^C of the extract and residues were 1330 ± 14‰ (mean ± SD) and 1313 ± 17‰, respectively. The δ^13^C of labeled needles was 1318‰. The C, N, P, and lignin concentrations in the needles were 477.1, 19.2, 1.51, and 312.6 g·kg^−1^, respectively. Each temperature group was split into four amendment treatments: (1) CF soil without litter (CF), (2) BF soil without litter (BF), (3) CF soil with litter (CFL), and (4) BF soil with litter (BFL). In the treatments with litter, the amount of added litter C was equal to 5% of the SOC content. Ground litter (<250 μm) was homogenously mixed with soil. The soil samples were incubated in 500 mL Mason jars (250 g fresh soil in each Mason jar) at two temperatures under aerobic conditions. Each treatment had three replicates (i.e., three Mason jars). Meanwhile, three Mason jars without soil samples were also incubated at each temperature. Vials (50 mL) without lids that contain 20 mL of 0.2 M NaOH solution were placed to trap respired CO_2_ in each Mason jar. Vials containing NaOH solution were taken after 1, 2, 6, 13, 21, 30, and 42 days and replaced by new NaOH vials. At the same time Mason jars were flushed with compressed air without CO_2_ for 20 min to replenish O_2_. Soil water content was measured by weighing each Mason jar every three days during the first 14 days and once a week after that, and deionized water was added to maintain moisture at 60% of field capacity.

To determine the δ^13^C of released CO_2_, 10 mL of NaOH solution was collected from the glass vial containing 20 mL of NaOH solution on each collection date. The δ^13^C of CO_2_ in the NaOH solution was measured using a stable isotope-ratio mass spectrometer^44^. The remaining 10 mL of NaOH solution was used to determine the amount of released CO_2_ using alkali-trapping techniques. The amount of CO_2_ was determined by titration with 0.1 M HCl to pH 8.3 after precipitation of carbonates with BaCl_2_, and calculated as the difference of the evolved CO_2_ from the Mason jars with and without soil.

### Partitioning CO_2_–C from litter and SOM sources

The amount of CO_2_–C measured by titration is the sum of litter and SOM decomposition. The application of ^13^C labeled litter allowed the partitioning of the total respired C into CO_2_ derived from litter and SOM. We assumed that isotopic fractionation of litter was negligible during litter decomposing. A mass balance equation was used to separate the amount of CO_2_–C derived from litter and SOM decomposition^40^:


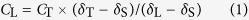



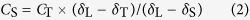


In Equations (1) and (2), *C*_T_ (*C*_T_ = *C*_L_ + *C*_S_) is the total amount of CO_2_–C during the considered time period, and *δ*_T_ is the corresponding value of δ^13^C. *C*_L_ is the amount of C derived from the added litter, and *δ*_L_ is δ^13^C of the litter. *C*_S_ is the amount of C derived from SOM, and *δ*_S_ is δ^13^C of SOM.

### Calculations of temperature sensitivity and priming effect

The temperature sensitivity (*Q*_10_) of litter and SOM decomposition was calculated by the following equation^9^:


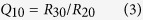


where *R*_20_ and *R*_30_ are respired CO_2_ rate at 20 and 30 °C, respectively.

The relative priming effect (PE, %) induced by the added litter was calculated by comparing the amount of CO_2_ derived from native SOM in the litter-containing soil samples with the amount of CO_2_ derived from native SOM in no-litter added soil samples^34^:


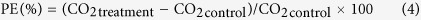


where CO_2treatment_ is the accumulated amount of CO_2_ derived from SOM in the treatments with litter addition, and CO_2control_ is the amount of CO_2_ derived from SOM without litter addition.

### PLFA determination

Soil microbial community composition was determined by PLFAs as biomarkers for different microbial groups. At the end of incubation, part of the soil was taken and immediately freeze-dried for PLFA analysis. Lipid extraction and PLFA analyses were performed based on the method described by White and Ringelberg^44^. Freeze-dried soil (5 g) was extracted using chloroform:methanol:phosphate buffer (1:2:0.8). The extracted PLFAs were purified on silica columns with chloroform, acetone, and methanol, amended with methyl-nonadecanoate as internal standard for quantifying the PLFAs, and converted to fatty acid methyl esters (FAMEs) through alkaline methanolysis. The concentration and isotopic composition of individual FAMEs was analyzed by gas chromatography–tandem mass spectrometry (Thermo Fisher). Qualitative standard mixes (37 Comp. FAME Mix and Bacterial Acid Methyl Esters CP Mix, Sigma-Aldrich) were used to identify the peak. Total bacterial content was calculated by summing i15:0, a15:0, 15:0, i16:0, 16:1ω7c, 16:1ω9c, 16:0, a17:0, i17:0, cy17:0, 17:0, 18:0, cy19:0, and 20:0 PLFAs^45^. Short- or odd-chain saturated PLFA (15:0, 16:0, 17:0, 18:0, and 20:0) were considered as common bacteria^46^, and PLFAs (i15:0, a15:0, i16:0, i17:0, and a17:0) were used as markers for the Gram-positive bacteria; PLFAs (16:1ω7c, 16:1ω9c, cy17:0, and cy19:0) were used as markers for the Gram-negative bacteria^25^. The 18:1ω9c, 18:1ω7c, and 18:2ω9,12c PLFAs were used as markers for fungi^45^. Mid-chain branched saturated PLFAs (10Me16:0, 10Me17:0, 10Me18:0) were associated with actinomycetes^46^.

The δ^13^C values of individual PLFAs were determined using isotope ratio mass spectrometry, as described by Moore‒Kucera and Dick^25^. The concentration (mg C kg^−1^ soil) of litter-derived C within PLFAs was calculated using the equations described by Nottingham *et al.*^41^


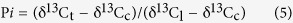


where δ^13^C_t_ is the δ^13^C enrichments (‰) of individual PLFA in the soils with leaf-litter at the end of incubation; δ^13^C_c_ is the δ^13^C enrichments (‰) of individual PLFA in the control soils; and δ^13^C_l_ is the δ^13^C of the labeled leaf litters (‰). The total labeled leaf-litter-derived C in each PLFA was calculated by multiplying each P*i* by individual PLFA abundances.

### Statistical analysis

The data were statistically analyzed using SPSS version 19.0 for Windows. The effect of litter addition on soil PLFA concentrations and the effect of temperature on the concentration of litter-derived C within different PLFA groups were tested by t-test after ANOVA. Significant differences in litter decomposition, *Q*_10_ values of SOM decomposition, and cumulative CO_2_-C from SOM among different treatments were tested by Tukey’s HSD after repeated measure ANOVA. ANOVA followed by Tukey HSD was performed to test significant differences in the concentration of litter-derived C within microbial groups and PE. Pearson correlation was performed to test the relationship between the PE and concentrations of microbial groups. *P* < 0.05 was considered significant.

## Additional Information

**How to cite this article**: Wang, Q. *et al.* Litter input decreased the response of soil organic matter decomposition to warming in two subtropical forest soils. *Sci. Rep.* **6**, 33814; doi: 10.1038/srep33814 (2016).

## Figures and Tables

**Table 1:** Changes in the concentrations (nmol g^−1^ soil) of PLFAs and two PLFA ratios in soils with needle addition at 20 and 30 °C at the end of 42-day incubation period.

		Common bacteria	GP	GN	GP/GN	Fungi	B/F	Actinomycete
20 °C	CF	9.25 ± 0.88aA	11.55 ± 1.09aA	5.97 ± 0.30aA	1.93 ± 0.12aA	3.88 ± 0.42aA	6.93 ± 0.39bA	2.94 ± 0.22aA
	CFL	11.51 ± 0.23bA	12.61 ± 0.41aA	7.34 ± 0.32bA	1.72 ± 0.13aA	6.00 ± 0.23bB	5.25 ± 0.21aA	3.48 ± 0.19aA
	BF	19.92 ± 0.13aA	18.27 ± 0.83aA	14.60 ± 1.42aA	1.26 ± 0.12aA	9.74 ± 0.39aA	5.42 ± 0.19bA	5.10 ± 0.29aA
	BFL	22.12 ± 0.76bA	17.37 ± 1.01aA	15.32 ± 0.54aA	1.13 ± 0.08aA	12.33 ± 0.51bA	4.45 ± 0.21aA	5.30 ± 0.26aA
30 °C	CF	9.25 ± 0.27aA	10.41 ± 0.34aA	5.74 ± 0.34aA	1.82 ± 0.11aA	3.28 ± 0.19aA	7.74 ± 0.29bA	2.98 ± 0.18aA
	CFL	11.73 ± 0.68bA	11.91 ± 0.46bA	6.75 ± 0.28bA	1.76 ± 0.12aA	4.51 ± 0.24bA	6.74 ± 0.32aB	3.33 ± 0.17aA
	BF	22.63 ± 0.95aB	19.08 ± 0.92aA	17.05 ± 0.84aA	1.12 ± 0.09aA	9.54 ± 0.55aA	6.17 ± 0.28aB	5.18 ± 0.20aA
	BFL	26.12 ± 0.45bB	21.76 ± 0.57bB	19.63 ± 1.27aB	1.11 ± 0.10aA	11.25 ± 0.61bA	6.01 ± 0.23aB	5.98 ± 0.55aA

Different lower case letters after data denote significant effect of litter addition on soil microbial community for same soils; different capital letters after data denote significant effect of temperature on soil microbial community for same soils. GP and GN represent Gram-positive and Gram-negative bacteria.

**Table 2:** Relationship between priming effect and concentrations of different microbial groups in CF and BF soils.

	Bacteria	Fungi	Bacteria:fungi	GP	GN	GP:GN	Actinomycete
CF	0.451	0.939**	−0.923**	0.630	0.781*	−0.492	0.377
BF	−0.940**	0.839*	−0.955**	−0.923**	0.898**	0.334	−0.648

*, **Denote significant difference at *P *< 0.05 and 0.01. GP and GN represent Gram-positive and Gram-negative bacteria.

**Table 3:** Properties of surface mineral soils in a coniferous forest (CF) and a broadleaved forest (BF).

	CF soil	BF soil
SOC (g·kg^−1^)	26.2 ± 2.3a	51.7 ± 1.2b
Total N (g·kg^−1^)	1.72 ± 0.20a	3.85 ± 0.13b
C:N ratio	15.2 ± 1.1b	13.4 ± 0.4a
Total P (g·kg^−1^)	0.20 ± 0.01a	0.35 ± 0.09b
NH_4_^+^-N (mg·kg^−1^)	4.77 ± 1.01a	8.46 ± 2.00b
Available P (mg·kg^−1^)	1.99 ± 0.45a	2.11 ± 0.59a
Sand content (%)	8.70 ± 1.91a	8.11 ± 1.73a
Silt content (%)	42.76 ± 1.30a	43.95 ± 1.29a
Clay content (%)	48.54 ± 1.95a	47.94 ± 2.01a

Different letters followed data in the same row denote significant difference at *P *< 0.05.
